# Isolated Gastric Varices due to Essential Thrombocytosis Related to Splenic Vein Thrombosis: A Challenge to Uncover the Concealed Diagnosis

**DOI:** 10.7759/cureus.6068

**Published:** 2019-11-04

**Authors:** Harish Patel, Peter Bhandari, Kishore Kumar, Jasbir Makker, Chaitanya Chandrala

**Affiliations:** 1 Internal Medicine, Bronx Lebanon Hospital Center, New York, USA; 2 Internal Medicine, American University of the Caribbean School of Medicine, Sint Maarteen, SXM; 3 Gastroenterology, Bronxcare Hospital Center, Bronx, USA; 4 Gastroenterology, Lake Erie College of Osteopathic Medicine, Elmira, USA

**Keywords:** isolated gastric fundus varices, splenic vein thrombosis, complications of essential thrombocytosis.

## Abstract

Essential thrombocytosis is associated with gastrointestinal (GI) vascular thrombosis. Sinistral portal hypertension (left-sided portal hypertension) due to splenic vein thrombosis can lead to isolated gastric varices and should be suspected in a patient with obscure GI bleeding with normal liver function. This case reviews the challenges presented in diagnosing isolated gastric varices due to splenic vein thrombosis through radiologic or endoscopic studies. Unrevealing radiologic or endoscopic studies does not rule out splenic vein thrombosis, which should be suspected in a patient with essential thrombocytosis and obscure GI bleeding.

## Introduction

Obscure gastrointestinal (GI) hemorrhage is defined as bleeding from an undiagnosed source after initial negative endoscopic and diagnostic evaluation [1]. The evaluation with esophagogastroduodenoscopy (EGD), colonoscopy, and small bowel follow-through is required to deem a GI bleeding etiology to be of obscure etiology. GI bleeding can be further identified as occult or overt based on the presentation. The manifestation with bright blood or altered heme product is overt GI bleeding.

Gastric varices are an identified etiology of GI bleeding, yet it may not be diagnosed on the initial EGD in a patient presenting with massive blood loss. Gastric varices can occur along with esophageal varices in patients with portal hypertension but isolated gastric varices occur in segmental portal hypertension like splenic vein thrombosis caused by various etiologies like pancreatic cancer, pancreatitis, pseudocyst, gastric cancer, renal cancer, splenic lymphoma and other hypercoagulable states [2]. Ultrasound vascular doppler has low sensitivity for diagnosis splenic vein thrombosis [3]. However, Doppler study remains the initial test for the evaluation of possible thrombosis; negative results are often not pursued for further evaluation. Initial unrevealing EGD and transabdominal splenic vein doppler do not rule out splenic vein thrombosis and require a high index of suspicion for further investigation.

We present a case of a patient with essential thrombocytosis and obscure-overt GI bleeding, who was diagnosed with gastric varices only after multiple presentations. Essential thrombocytosis is associated with gastro-intestinal vascular thrombosis [4]. Multiple Doppler studies of the splenic vein also failed to reveal any splenic vein thrombosis to explain the etiology of the isolated gastric varices. A confirmatory computed tomography (CT) scan with a celiac angiogram revealed the diagnosis of splenic vein thrombosis. Splenectomy is considered the optimal treatment for these patients as it eliminates collateral blood flow. For non-surgical candidates, splenic artery embolization in an alternative [5].

## Case presentation

A 65-year-old man presented initially to the emergency department (ED) with complaints of dizziness and passage of one episode of black-colored stool. The patient stated he had one episode of black-colored stool three days ago and the dizziness started the same day in the morning. The symptoms progressively worsened the last six hours and the patient presented to the ED. His medical co-morbid conditions were significant for diabetes mellitus and hypertension; his social history was significant for cigarette smoking (40 pack-years), but he denied alcohol or illegal drug use. His medication history included occasional use of low dose aspirin. Family history was significant for stomach cancer in the mother.

A physical exam revealed a pale and diaphoretic elderly man, with tachycardia and orthostatic hypotension. On presentation, the patient’s temperature was 37 C, heart rate was 120 beats per minute (bpm), respiratory rate was 18 breaths per minute, blood pressure was 104/64 mmHg, oxygen saturation in room air was 98%. The abdomen was soft, non-tender, non-distended with a palpable spleen. A rectal exam revealed melena.

Blood analysis revealed the patient to be anemic with hemoglobin of 5 gm/dL and hematocrit of 14.8. White blood cell count was 10.4 K cells/mcL, platelet count was 410 K cells/mcL. His comprehensive metabolic panel was within normal limits with serum sodium and potassium levels of 137 and 4.1 mEq/L, respectively. Blood urea nitrogen of 24 mg/dL and serum creatinine of 1.2 mg/dL. Protein levels were within normal limits with serum total protein of 6.4 g/dL and serum albumin of 3.6 g/dL. Liver enzymes were within normal limits with serum alanine transaminase and serum aspartate transaminase levels of 6 U/L and 9 U/L, respectively. Serum total and direct bilirubin levels were 0.3 mg/dL and 0.1 mg/dL. The international normalized ratio (INR) was 1.1. Serum lipase levels of 7 U/L were normal. Serum hepatitis B virus (HBV) surface antibody and hepatitis C virus (HCV) antibody were positive and negative, respectively. Serum thyroid-stimulating hormone was within normal limits with levels of 1.31 mIU/L. JAK II V617F mutation was detected indicating the presence of essential thrombocytosis.

Further diagnostic work-up

After adequate hemodynamic resuscitation with packed red cell transfusion with an interval increase of hemoglobin to 6.8 gm/ dL, he underwent upper GI endoscopy and colonoscopy. The patient was noted to have atrophic gastritis on upper endoscopy. Colonoscopy revealed diverticulosis of the sigmoid, transverse and terminal ileum. No active bleeding was noted. Further work-up for atrophic gastritis including anti intrinsic factor antibody and anti-parietal cell antibody was negative.

**Figure 1:**
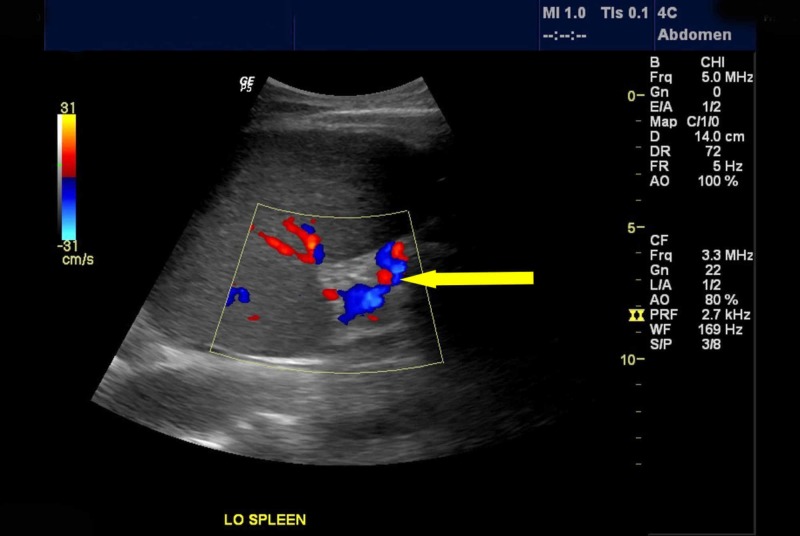
Ultrasound of the abdomen with splenic vein Doppler Longitudinal and transverse plane imaging shows borderline splenomegaly with no suggestion of splenic vein thrombosis. The arrow indicates vascular flow in the splenic vein.

The patient had a subsequent ED visit three months later and presented with melena and symptomatic anemia. The physical exam did not reveal any stigmata of liver cirrhosis. The patient underwent push enteroscopy, which revealed thickened gastric folds, with no signs of active bleeding in the visualized portions of the proximal and mid jejunum. During this admission, the patient was noted to have thrombocytosis with a platelet count of 787 x 106/mL. A further hematological evaluation revealed the diagnosis of essential thrombocytosis with a confirmatory JAK II V617F mutation. The patient was started on aspirin and low-dose hydroxyurea. The patient had no signs of liver failure or cirrhosis. To evaluate the etiology of the obscure GI bleeding, capsule endoscopy was done which revealed small amounts of blood in the stomach. A subsequent urgent upper GI endoscopy did not reveal any active bleeding and hence a Dieulafoy’s lesion was suspected. In view of worsening anemia, decreasing platelet count, and progressive leukocytosis, a bone marrow biopsy was done, which was negative for acute blast crisis. After stabilization, the patient was discharged with outpatient follow-up. A second look endoscopy was planned in the resuscitated phase with hemoglobin of 10.2 gm/dL. He was noted to have the isolated gastric varix in the cardia. The exam did not reveal any esophageal varices. The ultrasound of the abdomen with the splenic vein Doppler revealed a patent splenic vein (Figure 1).

**Figure 2:**
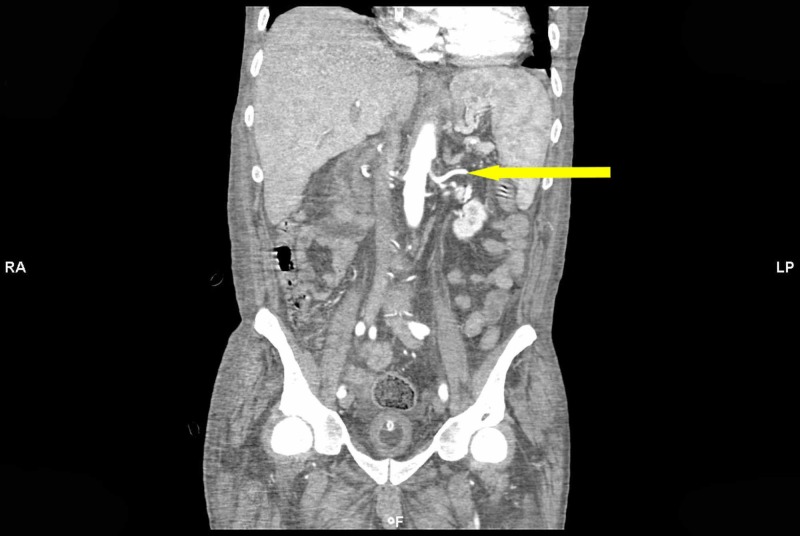
Computed tomography (CT) of the abdomen and pelvis with intravenous contrast Axial imaging shows an unremarkable spleen. The arrow indicates the splenic artery with no opacification of the portal system.

A month later, the patient presented to the ED again with melena. A repeat upper GI endoscopy showed isolated gastric varix in the cardia with a red sign but no active bleeding. The patient was treated with octreotide and proton pump inhibitors. Computed tomographic angiography (CTA) of the abdomen revealed varicosity in splenic hilum though no thrombosis could be identified (Figure 2). Radiologist opined poor opacification of the portal venous system. The patient was transferred to the tertiary care center for the splenic venogram and endoscopic management of gastric varices.

Final diagnosis and treatment

The final diagnosis of the presented case is isolated gastric varies secondary to splenic vein thrombosis in a patient with newly diagnosed essential thrombocytosis.

Various radiologic modalities and multiple endoscopic studies showed no evidence of splenic vein thrombosis in this patient. Due to a high index of suspicion for splenic vein thrombosis and after reviewing with radiology, the patient was transferred to a tertiary care liver center. A splenic venogram was performed at the liver center revealing splenic vein thrombosis. As the patient was deemed not a good surgical candidate for splenectomy due to thrombotic cardiovascular complications, he underwent splenic artery embolization by interventional radiology.

Outcome and follow-up

At subsequent follow-up visits to the clinic, the patient reported no further episodes of GI bleeding and the anemia had improved.

## Discussion

Obscure-overt GI bleeding is defined as evident bleeding without any recognizable source. The source of the bleeding cannot be identified in 5% of patients on initial evaluation with EGD and colonoscopy [6]. Amongst those with no identifiable source of bleeding in EGD and colonoscopy, small bowel etiology can be identified in 75% of cases. Small bowel evaluation with enteroscopy reveals a source of bleeding in 45% of the patients with obscure GI blood loss [7]. In patients younger than 40 years, small bowel tumor etiology is common while older individuals tend to have arterio-venous malformations [8]. For patients with recurrent bleeding and no identifiable etiology after upper, lower and small bowel work-up, a second look endoscopy is recommended [9].

Gastric fundic varices caused by obscure GI bleed is often overlooked. Based on location, isolated gastric varices can be classified into two types - IGV-1 (varices in fundus) and IGV-2 (isolated varices anywhere else in the stomach) [10]. Twenty percent of the patients with cirrhosis will have gastric varices, isolated or in association with the esophageal varices [11]. Patients with isolated gastric varices are more likely to have sinistral portal hypertension, (left-sided segmental portal hypertension) rather than liver cirrhosis [11-12]. In those patients who present with sinistral portal hypertension and absence of liver cirrhosis, a primary pancreatic etiology leading to splenic vein obstruction or thrombosis should be suspected [13]. Our patient did not have any evidence of liver cirrhosis and the imaging studies were unrevealing for any pancreatic pathology. However, the association of vascular thrombosis, in our patient, with essential thrombocytosis led us to pursue further work up for splenic vein thrombosis due to a high index of suspicion. We performed radiological imaging. However, the initial imaging test failed to reveal any thrombosis of the splenic vein.

Trans-abdominal splenic vein doppler is utilized as the initial test for the diagnosis of splenic vein thrombosis. However, splenic vein Doppler carries a low sensitivity in the diagnosis of splenic vein thrombosis as the flow in the collateral vessels near the splenic hilum could be interpreted as a patent splenic vein [14]. Hence, the presence of flow in peri-venous vasculature does not rule out splenic vein thrombosis [15]. Celiac angiogram is considered to be a confirmatory study for the diagnosis of splenic vein thrombosis [16-17]. There have been reports where splenic vein thrombosis is not diagnosed on ultrasound or celiac angiogram because of its proximal localization [18]. Rarely, surgical pathology reveals splenic vein thrombosis when other imaging modalities including angiogram fail to reveal the presence of thrombosis of splenic vein [17].

Endoscopic is considered to be the initial diagnostic modality for gastric varices. However, gastric varices can manifest as prominent gastric fold and pose difficulty in diagnosis [17]. Though there is no clear data, gastric varices can be flattened in view of the blood loss and hence may not be identified on the endoscopy. In our patient, on initial endoscopy, the varices were not visualized. However, EGD performed after adequate volume resuscitation (hemoglobin of above 10 gm/dL) revealed the gastric varices.

In patients with acutely bleeding gastric varices, hemostasis can be achieved with endoscopic variceal ligation [19]. It is well known that splenectomy can be performed in patients with bleeding gastric varices to prevent a further episode of bleeding. Splenectomy is considered to be optimal treatment due to the elimination of the collateral blood flow [5]. Patients with essential thrombocytosis undergoing splenectomy for essential thrombocytosis have a high risk for cardiovascular events [20]. Data regarding splenic artery embolization in a patient with bleeding gastric varices secondary to splenic vein thrombosis is limited. Hence in our patient after discussing the risks and benefits, splenic artery embolization was performed. Post-procedure follow-up revealed no further episodes of bleeding so far.

## Conclusions

Essential thrombocytosis is a common etiology of GI vascular thrombosis and can lead to splenic vein thrombosis. Isolated gastric varices, as a sequel of splenic vein thrombosis, is one of the etiologies of the obscure GI bleeding. Ultrasonography-assisted splenic vein Doppler is an initial modality for the evaluation of suspected splenic vein Doppler but carries a low sensitivity. There should be a high index of suspicion to pursue patients with splenic vein venogram to diagnose a case of splenic vein thrombosis.
